# President’s Address

**Published:** 1889-01

**Authors:** W. D. Haggard

**Affiliations:** Nashville, Tenn.


### Address.

### PRESIDENT'S ADDRESS.*

BY W. D. HAGGARD, M. D., NASHVILLE, TENN.

*Gentlemen of the Southern Surgical and Gynecological Association:*

Through your kindness I was chosen to preside over the deliberations of this learned assembly. I am grateful to my associates who have so honored me, though I fear it was your generosity, and not my fitness for the high and responsible position that prompted the selection, from so many more able and more distinguished members of the Association; but what I lack in ability, will, in some measure, be compensated by my enthusiasm in the cause. Feeling as I do, that the most capable and ardent among us must fall short of realizing the grand possibilities which await us, I have accepted the trust, with the mental reservation that, if nothing else, I am at least in earnest in what I say, and am filled with admiration and hope for the outcome of our labors. That I am proud of the privilege of addressing you I acknowledge, but I am prouder still of the occasion which calls forth the effort. I know you will all sustain me when I claim that gynecic surgery, with all its brilliant achievements, owes its present exalted position to the illustrious J. Marion Sims, no less than abdominal surgery owes its origin to the world-renowned Ephraim McDowell. The outcome of the labors of these two remarkable men in beneficent results have no parallel in the annals of medicine. They conferred on America the honor of being considered the birth-place of gynecology, and did more to alleviate the sufferings, restore the health, and prolong the lives, of women, than any two men living or dead.

*Read before the Sonthern Surgical and Gynecological Association, December 4th, 1888.

To those who knew Sims personally he gave inspiration while he lived, and now that he is dead he has become a tradition, and his teachings, his examples and his labors, have passed into history, and coupled his name with the practice of an art which will illuminate the ages to come. The first star he won in the galaxy of fame was his success in relieving vesico-vaginal fistula by the use of the silver wire suture, by which procedure thousands of women have been saved from a life of mortification and suffering far worse than death, restoring them to their families, their friends, and the social circle which they were so well calculated to adorn and beautify.

This achievement alone entitled him to the baptismal font of gynecology. From this point in his life's voyage his reputation spread with the rapidity and brilliancy of a meteor, alike illuminating this and distant lands. As one star outshines another, so it was given to J. Marion Sims to transcend all of his compeers in the glory of his achievements. He has written his name so high on the scroll of fame that the whole world may behold it, and so indelibly that all time cannot efface it, and to-day it is as familiar as household words with every true physician and surgeon on the globe. We deplore his loss, first, because we claimed him as our own, secondly, because he was in the fullness of a ripe experience and useful manhood; all too soon stricken down and taken from our midst, when, had he been spared to us, we might have had him here to-day to welcome you, instead of these poor words of mine.

May the light of his genius lend inspiration to us, and abide with us, and preside over our deliberations.

Addressing myself now more directly to those here assembled, many of whom, in the midst of urgent professional work, left their homes and loved ones to be present, and to participate in the proceedings of this Association, I now, in the name of the great State of Alabama, the brightest jewel that stands resplendent in the fair galaxy of States, once the home of the illustrious Sims, always the home of advanced thought in surgery and gynecology, I bid you welcome to this her city of destiny, and to the outstretched arms and open hospitality of the resident physicians and citizens of Birmingham, who greeted many of us on the occasion of our organization a little more than one year ago. I not only welcome you, but I take great pleasure in greeting you, one and all, as leading representatives of a noble and enlightened profession, whose object is to add to the span of human existence by preventing, alleviating and curing, the ailments incident to human life. The presence here of so many distinguished members of the profession, sustains the belief that our present deliberations will aid in advancing the already extensive and ever extending field of surgery and gynecology, and assist in unfolding the comp'ex problems ever pressing for solution in each of these departments. The aggregate benefit derived from this sort of professional intercourse is beyond description, but may be feebly outlined by stating that the formal preparation of scientific papers and reports on a great variety of subjects, necessitates a wide range of study and mental discipline, while the individual benefit is found in the collision with other minds in discussion, which brings out the salient points of the subject under review in all its varied aspects, thus enlarging the scope of mental vision, giving rise to new trains of thought, begetting a broader and stronger mental grasp; which is heightened by the presence of the great masters of the profession, at whose feet we sit and learn, and from whose magnetic influence, emanating from the kindly glance and the warm shake of the hand, we receive inspiration and take courage from the fact that we meet no more as strangers, but as brothers belonging to the great republic of letters, united in the bonds of mutual interest and affection, with purer and nobler aims. These advantages are so manifest, that at present a very large per cent. of the more advanced members of the profession throughout the world participate in organizations similar to ours. Is it too much to hope that the members of this organization are already united in bonds of love, which will grow with its growth and strengthen with its strength, until it receives for itself a domain of usefulness and a place of honor?

The Southern Surgical and Gynecological Association did not spring voluntarily into existence, but was the outgrowth of necessity, and is the embodiment of unknown power. It was the work of a few energetic minds and hearts, that saw its need and seized the opportunity to give it form and being, which was accomplished in this city, the second Tuesday in September 1887, at a most auspicious moment, and under most favorable circumstances. We offer to the profession an Association with which you may stand, without feeling that you are away from home; it embraces a declaration of principles to which you may unhesitatingly subscribe; its face is set in the right direction, with its eyes fixed upon the rising and not upon the setting sun. It forms a nucleus around which the votaries of science may gather, and as the great republic of letters forms the democracy of science, whose realms are penetrated by no royal road, but is open on all sides alike, to those who with patience, industry, energy and skill, shall ask to join the grand army of scientific observers, so we reach out our hands and invite into our circle, such men as by their work entitle them to the distinction of being educated, talented and industrious scientific workers in the fields of surgery and gynecology.

This Association has now met, for the first time since its organization, to lay the offerings of another year's work on the altar of science, and to further consider the present state of surgery and gynecology, and to contribute its mite in promoting the advancement of each department as a pure science, and their perfection as practical arts. None, perhaps, will question the supreme and commanding fact, that in these two branches of medical science the greatest advances have been made. To those of us who can look back on a decade of professional life, the improvement that has taken place in that length of time is very gratifying, and inspires a feeling of hopefulness for the future. As much yet remains to be accomplished, we can enter cheerfully upon the work we have undertaken as cultivators of the science and the arts of surgery and gynecology. We are now young and vigorous, let us work in the hey-day of youth, that we may keep and add to our strength and influence. Long may this and kindred Associations last, and may our own prove not the least worthy and efficient of them all. The trend toward specialism, already strong, is continually increasing, and while there is danger that those giving themselves up to special lines of work may become deficient in the fundamental principles of medicine as a whole, there are cogent reasons impelling the movement. The advent of Sims specially marked an epoch of advancement in this direction, by clearing away many false though ingenious theories and dogmas, which were obscured by the fogs of dissension, leaving them to rest in the sun-light of truth.

But, gentlemen of the Association, I am admonished by the number and character of the papers which appear on the program, as well as by the marked ability of those present who are likely to take part in the discussions, that the time of the Association will be so fully occupied, that I have presumed on your approval of the omission of an address on some scientific subject, but may have, in its stead, to ask your indulgence in debate as well as profit by the discussions.

In declaring the Association now regularly opened for the transaction of business, I must ask your indulgence and generous forbearance with my slender ability in the discharge of the responsible duties which devolve upon me, as your presiding officer.

---

Much of the success of the recent meeting of the Southern Surgical and Gynecological Association, was due to the untiring efforts of Drs. J. D. S. and W. E. B. Davis, two of Birmingham's most popular and progressive physicians.

